# Association between new-onset Parkinson’s disease and suicide risk in South Korea: a nationwide cohort study

**DOI:** 10.1186/s12888-022-03990-4

**Published:** 2022-05-17

**Authors:** Sung Hoon Jeong, Seung Hoon Kim, Doo Woong Lee, Eun-Cheol Park, Suk-Yong Jang

**Affiliations:** 1 Department of Public Health, Graduate School, Yonsei University, Seoul, Republic of Korea; 2 Institute of Health Services Research, Yonsei University, Seoul, Republic of Korea; 3 Department of Preventive Medicine, Yonsei University College of Medicine, Seoul, Republic of Korea; 4 Department of Healthcare Management, Graduate School of Public Health, Yonsei University, 50-1 Yonsei-ro, Seodaemun-gu, Seoul, 03722 Republic of Korea

**Keywords:** Suicide, Risk, Parkinson’s disease, Psychiatric disorder, Depression

## Abstract

**Background:**

Parkinson’s disease (PD) is an increasingly common neurodegenerative disease in an aging society. Whether PD is associated with an increased suicide risk is unclear. Thus, we investigated the effect of new-onset PD on suicide.

**Methods:**

Using the National Health Insurance Service Senior Sample Cohort of South Korea, 17,143 incident PD patients and 17,143 risk set controls, matched by propensity score, were selected for follow-up. The incidence rate of suicide and 95% confidence interval (CI) were calculated based on a generalized linear model of the Poisson distribution. Effect sizes were expressed as hazard ratios (HRs) using the Cox proportional hazards model with a robust variance estimator that incorporated clustering within matched pairs.

**Results:**

The incidence rate of suicide was 206.7 cases per 100,000 person-years (95% CI, 172.8–246.9) among the PD cohort. Compared to the matched controls, patients with PD were 2.64 times (HR, 2.64; 95% CI, 1.31–5.30) more likely to commit suicide during the first 180 days of follow-up and 2.47 times (HR, 2.47; 95% CI, 1.42–4.28) within the first 365 days of follow-up. During the entire follow-up period, patients with PD were 2.26 times more likely to commit suicide than were their matched controls (HR, 2.26; 95% CI, 1.67–3.06).

**Conclusion:**

Our findings indicated an increased risk of suicide in patients with new-onset PD, regardless of the period after diagnosis. Incorporating mental health care with social and environmental interventions into primary care and PD-specialized care can help reduce suicide risk in people with PD, improving suicide prevention, identification, and risk assessment.

**Supplementary Information:**

The online version contains supplementary material available at 10.1186/s12888-022-03990-4.

## Background

Suicide, defined as the act of deliberately killing oneself, is considered a major public health problem in many countries [1]. The World Health Organization (WHO) reported that approximately 804,000 people died by suicide in 2012, and by 2020 that number is expected to reach 1.5 million [2, 3]. Older people, in particular, are at a higher risk of suicide worldwide than other age groups, and the number of suicides is expected to increase over the next few decades as the population ages rapidly [4, 5]. Therefore, it is important to target the elderly to reduce the burden of suicide.

Parkinson’s disease (PD) is the second most common neurodegenerative disease, after Alzheimer’s disease, among people over the age of 60, and its prevalence increases with age [6, 7]. Motor symptoms and psychiatric symptoms such as depression and anxiety are common during the course of PD [8]. Approximately 40%–50% of people with PD are affected by depression and 30%–40% by anxiety disorders such as generalized anxiety, social phobia, and panic attacks [8–10]. In addition to the long-established link between psychiatric disorders and suicide [11], the physical limitations caused by PD and the increased risk of suicide in the elderly require attention to the suicidal potential of people with PD [4].

Several studies have investigated the association between PD and suicide but have not provided consistent results. Several studies have demonstrated a higher suicide rate among people with PD [12–14], whereas other studies have demonstrated the opposite finding [15–17]. One study [18] also reported a lower suicide risk in patients with PD than in the general population. Given the increasing suicide rate among the elderly, studies using a large representative population-based PD sample are needed to determine reliable estimates of suicide rates.

The suicide incidence rate (IR) in patients with PD has not been previously investigated. Therefore, in this study, the suicide IR was evaluated by using a national cohort. The second purpose of this study was to analyze the hazard ratio (HR) of suicide over time among patients with PD after the onset of PD, compared to that of the control group, who were matched with propensity score matching.

## Methods

### Data collection and participants

Parkinson’s patients and their corresponding controls were selected from the National Health Insurance Service Senior Cohort (NHIS-Senior) of Korea. The NHIS established the National Health Information Database (NHID), a claims database for research purposes that stores all records of healthcare and long-term care services [19]. Based on the NHID data, the NHIS constructs and provides researchers with the NHIS-Senior, a representative administrative data set used for health policy and biomedical research purposes [19]. NHIS-Senior consists of 558,147 people selected in 2002 using a 10% simple randomization method from a total of 5.5 million individuals aged ≥ 60 years [19]. Under the compulsory social insurance system enacted under the National Health Insurance Act, all targets, except death or immigration, can be tracked until 2013 [19, 20]. The National Health Insurance Corporation keeps all personal information, demographic data, and medical data about the entire population of Korea [19, 20]. Key variables for NHIS-Senior include inpatient and outpatient billing data such as treatment procedure codes, prescriptions, and diagnoses [20].

All data are available in the database of the Korean National Health Insurance Sharing Service (https://nhiss.nhis.or.kr) and can be accessed upon reasonable request. The study protocol was approved by the Institutional Review Board of Yonsei University Severance Hospital (approval no: 4–2021-1211; Seoul, Republic of Korea). Due to the retrospective nature of the study, the requirement to obtain informed consent was waived.

### Incident PD cohort

The incident PD cohort was constructed from the NHIS-Senior base cohort. To be enrolled, individuals must fit the operational definition of new-onset PD, such as admission to an acute care hospital or two or more outpatient visits with a primary or the first secondary diagnostic code of PD (International Statistical Classification of Diseases and Related Health Problems, 10th Revision [ICD-10] G20). Several previous studies in Korea were referred to for the definition of the ICD-10 code for the PD patient group [21, 22]. To select new-onset PD cases, patients with a medical claim for PD in 2002 or 2003 (i.e., a 2-year washout period) were excluded because they had a previous diagnosis of PD. The PD onset date was defined as the first hospitalization or second outpatient visit, whichever occurred first. The second outpatient visit was considered the date of PD diagnosis to avoid an immortal time bias [23], which is the interval between the first and second outpatient visits [24]. Thus, beginning on January 1, 2004, patients with new-onset PD were enrolled and followed up in the event PD cohort on the index date (i.e., time zero).

### Identification of suicide cases

Based on Korean law, all death certificates must be reported to the National Statistical Office (Seoul, Republic of Korea); therefore, the National Statistical Office collects information on death (i.e., date and cause) and links the data individually by using a unique personal identification number [19, 20]. Suicide has been identified as a series of deaths classified as "intentional self-harm" (ICD-10 codes X60–X84). The time of the event was defined as the date of death by suicide [1, 19, 20].

### Risk set matching on propensity score

The National Health Insurance Service Senior–National Sample Cohort (NHIS-NSC) was constructed retrospectively, although this study was designed to mimic a prospective study. Therefore, here we solved many limitations inherent to retrospective design through risk-set matching with time-dependent propensity scores [23, 25]. First, the association between PD and suicide risk was observed with time-dependent propensity score matching to adjust for confounding factors [26]. As with the propensity score, we estimated the risk factors in the Cox proportional hazards model with January 1, 2004 as the baseline and the PD event [26]. Therefore, values ​​collected during the two years prior to baseline (2002–2003) were included as covariates (Table 1). Specifically, age was included as a continuous variable, and gender, household income level, region, social security, enrollment disability, Charlson Comorbidity Index (CCI), and medical history were included as categorical variables. The number of comorbidities in each individual was assessed as a diagnostic code by using the Quan ICD-10 coding algorithm of CCI scores [27]. Patients who were prescribed antihypertensive, antidiabetic, lipid-lowering, and antidepressive agents for more than 90 days were considered to use the drug [23]. Second, to mimic a prospective study, when a patient with PD was first identified, individuals at risk of PD but not yet at onset were matched among patients of the same age and sex as the identified patient with PD. Next, in the case of the matched control group, the diagnosis date of the matched PA patient was set as the index date (time 0), and the progress was observed. Furthermore, we repeated this risk-set matching method for all subsequent PD patients [28–30]. Finally, 1:1 matching on propensity scores was sequentially conducted for each risk set by using the nearest neighbor matching algorithm with a maximum caliber of 0.1. To ensure that the match was independent of future events, the matched control subjects could either be those who had never developed or were yet to develop PD. Most of the patients who had PD were included in the incident PD cohort. However, some patients with PD could be included as matched controls because they were selected as matched control subjects before PD onset [31]. Next, the matching patients were removed from the next set of risks to generate non-overlapping samples from the set of risks. This process was repeated for the next risk set until no treated patients were in the risk set.

**Table 1:** Baseline characteristics of Parkinson's disease patients and their risk set-matched cohort

Variables	Total			Parkinson’s disease (*N* = 17,143)		Matched Control (*N* = 17,143)		Standardized Difference
**Total**	34,286		(100.0)	17,143	(50.0)	17,143	(50.0)	
**Sex**								0
Male	12,922		(37.7)	6,461	(37.7)	6,461	(37.7)	
Female	21,364		(62.3)	10,682	(62.3)	10,682	(62.3)	
**Age (Mean, SD)**	71.10		6.02	71.10	6.02	71.10	6.02	0
**Household income level**								0.0143
Low	9,428		(27.5)	4,767	(27.8)	4,661	(27.2)	
Mid	9,865		(28.8)	4,924	(28.7)	4,941	(28.8)	
High	14,993		(43.7)	7,452	(43.5)	7,541	(44.0)	
**Region**								0.0047
Metropolitan	11,025		(32.2)	5,515	(32.2)	5,510	(32.1)	
City	7,457		(21.7)	3,743	(21.8)	3,714	(21.7)	
Rural	15,804		(46.1)	7,885	(46.0)	7,919	(46.2)	
**Social Security**								0.0158
Insurance (Regional)	11,743		(34.3)	5,935	(34.6)	5,808	(33.9)	
Insurance (Corporate)	17,853		(52.1)	8,883	(51.8)	8,970	(52.3)	
Medical Aid	4,690		(13.7)	2,325	(13.6)	2,365	(13.8)	
**Disability**								0.0124
No	33,978		(99.1)	16,979	(99.0)	16,999	(99.2)	
Yes	308		(0.9)	164	(1.0)	144	(0.8)	
**Charlson Comorbidity Index (CCI)**								0.0156
0	18,583		(54.2)	9,285	(54.2)	9,298	(54.2)	
1	8,350		(24.4)	4,136	(24.1)	4,214	(24.6)	
2	3,939		(11.5)	1,984	(11.6)	1,955	(11.4)	
≥ 3	3,414		(10.0)	1,738	(10.1)	1,676	(9.8)	
**Medication history**								
** Antidiabetic agents**								0.0037
No	29,540		(86.2)	14,759	(86.1)	14,871	(86.7)	
Yes	4,746		(13.8)	2,384	(13.9)	2,362	(13.8)	
** Antihypertensive agents**								-0.0042
No	20,327		(59.3)	10,181	(59.4)	10,146	(59.2)	
Yes	13,959		(40.7)	6,962	(40.6)	6,997	(40.8)	
** Lipid-lowering agents**								0.0409
No	31,319		(91.3)	15,561	(90.8)	15,758	(91.9)	
Yes	2,967		(8.7)	1,582	(9.2)	1,385	(8.1)	
** Antidepressive agents**								0.0016
No	30,211		(88.1)	15,101	(88.1)	15,110	(88.1)	
Yes	4,075		(11.9)	2,042	(11.9)	2,033	(11.9)	
**Medication history**								
** Malignant neoplasm**								0.0221
No	33,307		(97.1)	16,622	(97.0)	16,685	(97.3)	
Yes	979		(2.9)	521	(3.0)	458	(2.7)	
** Ischemic heart disease**								0.0120
No	31,545		(92.0)	15,707	(91.6)	15,838	(92.4)	
Yes	2,741		(8.0)	1,436	(8.4)	1,305	(7.6)	
** Stroke**								0.0282
No	32,478		(94.7)	16,216	(94.6)	16,262	(94.9)	
Yes	1,808		(5.3)	927	(5.4)	881	(5.1)	

Values are presented as mean ± standard deviation or number (%)

At the date of Parkinson’s disease incidence of each patient, two controls were matched on follow-up time and propensity score estimated by Cox proportional hazards model with predictors included in this table

### Statistical analyses

Statistical tests of the association between incident PD and suicide risk were conducted while considering the statistical nature of matched-pair analysis using the final matched cohorts. To assess the covariate balance between treatment groups, baseline characteristics were compared to standardized differences in which differences < 0.1 (10%) were generally considered negligible [32, 33]. The effects of PD on suicide risk were considered relatively severe in this study. Therefore, in addition to the overall period, several periods (180 days, 365 days, and 730 days) were analyzed as follow-up periods in the survival analysis. The cumulative incidence curves of suicide were obtained as Kaplan–Meier survival curves, and a stratified log-rank test was used to compare Kaplan–Meier curves of matched cohorts [34]. The cumulative incidence and 95% confidence intervals (CIs) of suicide were calculated by the product-limit (Kaplan–Meier) method of survival probabilities. The IR (95% CI) of suicides was calculated based on a generalized linear model of the Poisson distribution and was expressed as the number of suicides per 100,000 person-years. Effect sizes were expressed as the HR, using a Cox proportional hazards model with a robust variance estimator that accounts for clustering within matched pairs [33, 34]. Time zero was established as the date of onset of PD for patients with PD and their matched controls. Survival time used in the analysis was defined as months from time zero of death or December 31, 2013, whichever came first. In addition, subgroup analyses within categorized age groups (60–69 years and ≥ 70 years), sex, and region (metropolitan, city, rural) were conducted. Differences with *p* < 0.05 were statistically significant. All data analyses were conducted using SAS 9.4 (SAS Institute Inc., Cary, NC, USA).

## Results

### Flow of analysis and the baseline characteristics of patients

**Fig. 1:**
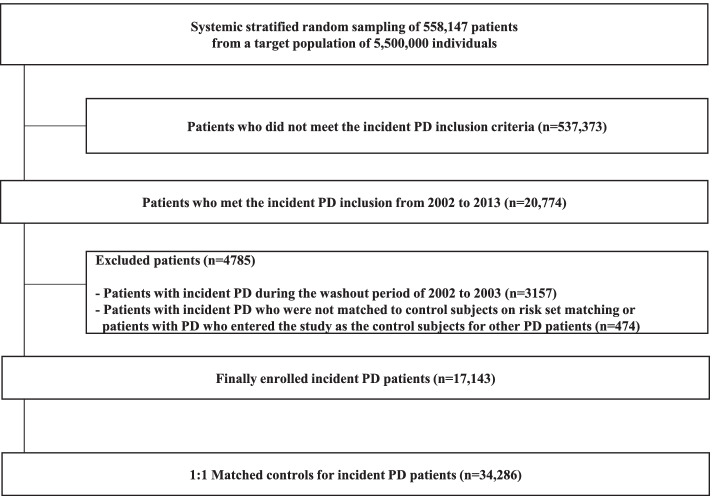
Study flow and disposition of the patients with PD. PD, Parkinson’s disease

From January 1, 2002 to December 31, 2013, a total of 20,774 patients met the inclusion criteria. Among these patients, 3157 patients with PD during the washout period from 2002 to 2003 were excluded. During risk set matching, patients with PD who did not match controls or another patient who entered the study as controls for patients with other PD were excluded (474 patients). Finally, 17,143 incident PD patients and 17,143 matched controls were analyzed in this study (Fig. 1). Table 1 shows the baseline characteristics of the matched cohorts. The mean age was 71.1 years, and 62.3% of the patients were female. A total of 185 suicide cases were identified in follow-up.

### Suicide risk in PD

**Fig. 2:**
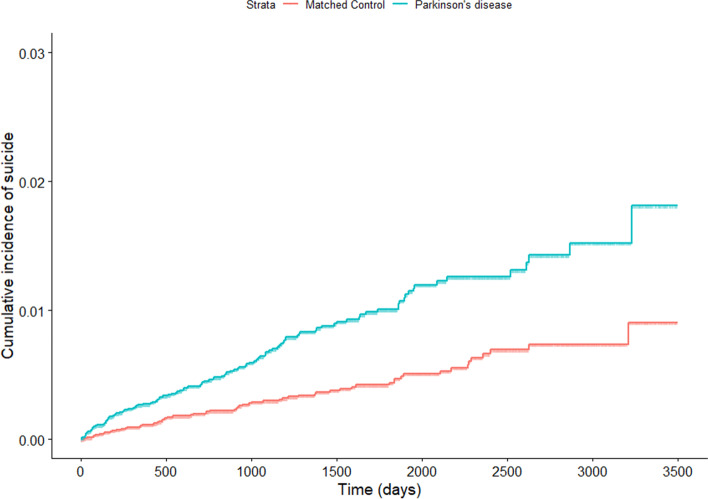
Comparison of cumulative incidence of suicide between incident PD patients and their risk set-matched controls. *P*-value for stratified log-rank test < 0.0001. PD, Parkinson’s disease

Cumulative incidence of suicide during the entire follow-up period showed a statistically significant difference between PD and the matched-control cohort (*p* < 0.0001 for log-rank test) (Fig. 2).

During the entire follow-up period, 121 suicides were confirmed during 58,530.1 person-years (IR, 206.7 per 100,000 person-years) of 17,143 patients with PD. When all covariates were adjusted for, over the whole period, patients with PD were 2.26 times more likely to commit suicide than matched controls (HR, 2.26; 95% CI, 1.67–3.06) (Table 2; Supplementary Table 1). During the first 180 days of follow-up, 28 suicides were confirmed during 7912.5 person-years (IR, 353.9 per 100,000 person-years) of 17,143 patients with PD. Patients with PD were 2.64 times more likely to die by suicide than controls over the same period (HR, 2.64; 95% CI, 1.31–5.30). During the first 365 days of follow-up, 42 suicides were confirmed during 15,135.1 person-years (IR, 277.5 per 100,000 person-years) of 17,143 patients with PD. Patients with PD were 2.47 times more likely to die by suicide than controls over the same period (HR, 2.47; 95% CI, 1.42–4.28) (Table 2).

**Table 2:** Comparable analysis of suicide rate for the association between Parkinson’s disease and risk of suicide

Expos ure	No. of subjects	No. of suicide	Person_year	Incidence rate (95% CI) per 100,000 person-years	95% CI	Crude HR	95% CI	Adjusted HR^*^	95% CI
**0–180 days**									
Matched control	17,143	11	8151.3	134.9	(74.3–242.3)	1.00			
Parkinson patients	17,143	28	7912.5	353.9	(242.9–509.5)	2.62	(1.30–5.26)	2.64	(1.31–5.30)
**181–365 days**									
Matched control	15,870	7	7711.8	90.8	(43.0–189.3)	1.00			
Parkinson patients	15,039	14	7200.8	194.4	(114.5–326.4)	2.14	(0.86–5.31)	2.19	(0.88–5.43)
**0–365 days**									
Matched control	17,143	18	15,880.5	113.3	(71.2–179.4)	1.00			
Parkinson patients	17,143	42	15,135.1	277.5	(204.4–374.4)	2.43	(1.40–4.23)	2.47	(1.42–4.28)
**366–730 days**									
Matched control	14,625	11	13,396.7	82.1	(45.3–147.8)	1.00			
Parkinson patients	13,484	21	12,077.6	173.9	(113.0–265.9)	2.12	(1.02–4.39)	2.11	(1.02–4.39)
** > 730 days**									
Matched control	12,162	35	39,688.6	88.2	(63.3–122.7)	1.00			
Parkinson patients	10,713	58	31,317.4	185.2	(143.0–239.4)	2.08	(1.37–3.17)	2.19	(1.44–3.33)
**Full cohort**									
Matched control	17,143	64	68,965.8	92.8	(72.6–118.5)	1.00			
Parkinson patients	17,143	121	58,530.1	206.7	(172.8–246.9)	2.19	(1.62–2.97)	2.26	(1.67–3.06)

*CI* confidence interval

*Adjusted for other covariates

### Subgroup analysis for suicide risk, based on age, sex, and region

In a subgroup analysis, female patients with PD had a 2.74 times higher risk of suicide (HR, 2.74; 95% CI, 1.72–4.37), and patients with PD aged 60–69 years had a 3.19 times higher risk of suicide (HR, 3.19; 95% CI, 1.87–5.46). City resident patients with PD had a 4.93-fold higher risk of suicide (HR, 4.93; 95% CI, 2.27–10.69). Furthermore, among those who had used antidepressants, the risk of suicide was 2.61 times higher in patients with PD (HR, 2.61; 95% CI, 1.37–4.95) (Table 3).

**Table 3:** Analyses of the association between Parkinson's disease and risk of Suicide stratified by independent variables

Variables		Risk of Suicide	
		Adjusted HR^*^	**95% CI**
**Sex**			
** Male**	1.00	1.96	(1.31–2.93)
Female	1.00	2.74	(1.72–4.37)
**Age**			
** 60–69**	1.00	3.19	(1.87–5.46)
** 70-**	1.00	1.88	(1.29–2.73)
**Region**			
Metropolitan	1.00	1.69	(1.02–2.80)
City	1.00	4.93	(2.27–10.69)
Rural	1.00	2.06	(1.31–3.23)
**Antidepressive agents**			
No	1.00	2.18	(1.55–3.08)
Yes	1.00	2.61	(1.37–4.95)

^*^Adjusted for other covariates

## Discussion

After adjusting for multiple confounders in a large national representative population-based cohort, the risk of suicide in patients with PD was approximately twice that of the general population. The main results of this study are as follows. First, the suicide rates of elderly patients with PD were 206.7/100,000 person-years within the entire follow-up period of this study, 353.9/100,000 person-years within 180 days, and 277/100,000 person-years within 1 year. Second, elderly patients with PD were 2.19 times more likely to die from suicide within the entire follow-up period of this study, 2.64 times more likely to die from suicide within 180 days, and 2.43 times more likely to die from suicide within 1 year. Furthermore, in the PD cohort, we found that the risk of suicide was constant and persistent over time. No specific high- or low-risk periods have been identified. In contrast to Alzheimer’s disease, where a high risk for suicide is usually found early and then decreases [35, 36], our study observed persistent maintenance of suicide risk in PD, as in studies in Denmark and Taiwan [4, 35].

Suicide in the elderly is a major health problem in developed countries [13]. Several studies have already reported a high suicide rate among older people in many countries [37]. In this study, the remarkably high suicide rate of 206.7 per 100,000 person-years in patients with PD is associated with the steadily increasing number of elderly suicides in South Korea; in 2010, it was reported to be 80 per 100,000 [38]. In a study of suicide rates due to physical illness in patients over the age of 65 in Denmark, the rates were 43.7 per 100,000 person-years for men and 18.7 per 100,000 person-years for women [14]. In addition, the suicide rate of lung cancer patients in the United States was reported to be 27.5 per 100,000 person-years [39], and the suicide rates of cancer survivors in the United States and South Korea were 31.4 and 88.7 per 100,000 person-years, respectively [40]. When comparing suicide rates with these various physical ailments, the results of this study cannot be ignored.

Some previous studies' findings conflict with those reported here and show that there is no association between PD patients and suicide risk; the differences in study design may explain the contrasting results. Specifically, in the case of studies showing no association between PD patients and suicide risk, the control group was not comparable to the PD group in terms of demographic and health-related characteristics, the time as a variable was not considered [15–18], and the number of suicide events was not sufficient to examine the association between PD and suicide [15, 17]. By supplementing these limitations with propensity scores matching and the risk-set matching methodology, our estimate that Parkinson's patients have an approximately doubled risk of suicide is similar to the findings of Danish studies in 2015 (men: HR, 1.92 [95% CI, 1.26–2.92]; women: HR, 2.26 [95% CI, 1.25–4.08]) and 2020 (HR, 1.7 [95% CI, 1.5–1.9]) [14, 35], as well as the UK (adjusted odds ratio, 2.41 [95% CI, 1.15–5.07]) [41] and Taiwan (HR, 1.9 [95% CI, 1.6–2.3]) [4].

The association between PD and suicide can be explained through several mechanisms. First, being diagnosed with PD may constitute acute life events that are prominent risk factors for suicide [42]. Second, depression, the most prominent risk factor for suicide, is a common nonmotor symptom in PD [8, 9]. Third, functional decline, a common characteristic of PD, has been suggested as an important contributing factor to suicide in the elderly [43]. Functional impairment can lead to disability, dependence, perceived burden, and disconnection from social networks. These are all risk factors for suicide in old age [43]. Finally, the suicide risk of PD may appear with treatment. Although inconsistent, some studies have reported an association between dopaminergic agonists and suicide in patients with PD [12]. However, other studies have not supported this association [4, 44]. Additionally, although some reports have suggested an increased risk of suicide in patients with PD and subthalamic nucleus stimulation, the association remains unclear [45, 46].

Our study also found that women, younger people, city residents, or patients with PD taking antidepressant medications were significantly affected by suicide. This result in women may be because in female patients with PD, motor disorders and depression, which are highly correlated with suicide, are commonly observed [47]. Furthermore, in a preliminary study of patients with PD, it was observed that quality of life was significantly lower in women [48].

The influence on suicide in younger patients with PD is probably because motor fluctuations occur more frequently in younger patients with PD. Motor fluctuations can negatively affect relatively young and cognitively sound patients, leading to suicide [13, 49]. It is consistent with many previous studies that patients with PD residing in cities are highly affected by suicide [4]. This is because it is easy for frail elderly people who live in high-rise buildings in urban areas to jump out of their homes, and these methods are easily accessible [50, 51]. In fact, in Korea, people in urban areas were twice as likely to jump from high places and die as were people in rural areas [52]. Nevertheless, caution should be exercised in these interpretations as they may be based on cultural issues. Additionally, several studies have demonstrated that depression in elderly patients with PD is strongly associated with suicide [53]. Therefore, it can be considered that the suicide of patients with PD taking antidepressant drugs was greatly influenced by the synergistic effect of depression and PD itself on suicide.

Our study has several strengths. First, we were able to conduct a well-represented study using nationwide insurance claim data. Because the study was conducted with the entire population and was not limited to a specific hospital or institution, it can be considered that the enrolled study subjects are representative of the entire population without bias. Second, we also investigated the association between PD and suicide risk with risk-set matching using a nationwide cohort based on insurance claims, which is currently one of the best available methods. Risk-set matching enables an analysis similar to that of randomized clinical trials in observational studies when the former cannot be performed; specifically, this method is one of the most important ways to reduce immortal time bias in epidemiological studies [24, 54]. Therefore, at-risk patients were matched by propensity scores when Parkinson's patients were identified, and changes in covariates after time 0 were not considered to avoid immortal time bias.Third, because the patients enrolled in this study were tracked using insurance claim data due to the nature of the study, there was no tracking loss except for rare immigration or detention. Lastly, unlike previous studies, this study focused on suicide IR of elderly patients with PD and presented suicide HR over time after PD diagnosis.

There are some inherent limitations to our study. First, an accurate diagnosis of PD based on the use of insurance claim data may not be accurate. A wide range of diagnostic codes can be added to the patient's medical examinations, treatment modalities, and drug prescriptions in the course of patient care in a hospital or clinic. To overcome these limitations, this study used not only the two earliest diagnoses, but also patients who were admitted more than twice as outpatients and more than once for hospitalization. Second, the clinical information of this study is limited because it is based on insurance claim data. A further limitation was that certain clinical information, such as blood test results, was not included. Third, since this is a study related to suicide in patients PD, it is possible that confounding variables such as education level, drinking, smoking, dyskinesia, and low morale may affect the incidence of PD and suicide [55, 56]. In addition, due to lack of information, some important variables, such as the severity of PD, were not included in the model for calculating the propensity score. To overcome these pitfalls, we used propensity scores to select and use 1:1 concordant cohorts. Nevertheless, residual confounding factors may exist after propensity score matching. Therefore, we tried to overcome these shortcomings through Cox regression analysis considering socio-economic indicators such as CCI score and income level.

## Conclusion

Our study found approximately two times increased risk of suicide in patients with new-onset PD, regardless of the duration after diagnosis. Patients diagnosed with PD are accompanied by psychological distress such as depression as well as physical signs such as decreased motor performance. Incorporating mental health care with social and environmental interventions into primary care and PD-specialized care can help reduce suicide risk in people with PD, improving suicide prevention, identification, and risk assessment.

## Supplementary Information

**Additional file 1:** **Supplementary Table 1. **Results of the associationbetween Parkinson's disease and the risk of suicide. 

## Data Availability

The Korean National Health Insurance Service–National Sample Cohort is a public, open-access database. It is based on the health insurance claim data of all Koreans, and the sample cohort is available for public purposes and scientific research. The authors do not have permission to share these data. The sample cohort data are available after acceptance of approval for use by the national health insurance service. (https://nhiss.nhis.or.kr/bd/ab/bdaba000eng.do).

## Abbreviations

CCI: Charlson Comorbidity Index
CI: Confidence interval
HR: Hazard ratio
IR: Incidence rate
NHID: National Health Information Database
NHIS: National Health Insurance Service Senior
PD: Parkinson’s disease
WHO: World Health Organization
