# Supplementary material for: Association between new-onset Parkinson’s disease and suicide risk in South Korea: a nationwide cohort study
Source: BMC Psychiatry. 2022 May 17;22:341. doi: 10.1186/s12888-022-03990-4 (PMC9115980; doi:10.1186/s12888-022-03990-4)
Supplement: Supplementary file 1 — Additional file 1: Supplementary Table 1. Results of the associationbetween Parkinson's disease and the risk of suicide. [file 12888_2022_3990_MOESM1_ESM.docx]

| **Supplementary Table 1. Results of the association between Parkinson's disease and the risk of suicide** | | | | | |
| --- | --- | --- | --- | --- | --- |
|  |  | **Risk of Suicide** | | | |
| **Variables** | | **HR** | **95% CI** | | |
| **Parkinson's disease** | |  |  |  |  |
| **No** | | **1.00** |  |  |  |
| Yes | | 2.26 | (1.67 | - | 3.06) |
| **Sex** | |  |  |  |  |
| Male | | **1.00** |  |  |  |
| **Female** | | 0.43 | (0.32 | - | 0.58) |
| **Age (per 1 year)** | | 1.02 | (1.00 | - | 1.05) |
| **Household income level** | |  |  |  |  |
| Low | | **1.00** |  |  |  |
| Mid | | 0.67 | (0.43 | - | 1.06) |
| High | | 0.68 | (0.44 | - | 1.03) |
| **Region** | |  |  |  |  |
| Metropolitan | | **1.00** |  |  |  |
| City | | 0.98 | (0.66 | - | 1.46) |
| Rural | | 0.92 | (0.66 | - | 1.28) |
| **Social Security** | |  |  |  |  |
| Insurance (Regional) | | **1.00** |  |  |  |
| Insurance (Corporate) | | 1.48 | (1.05 | - | 2.07) |
| Medical Aid | | 1.21 | (0.68 | - | 2.16) |
| **Disability** | |  |  |  |  |
| No | | **1.00** |  |  |  |
| Yes | | 0.88 | (0.22 | - | 3.55) |
| **Charlson Comorbidity Index (CCI)** | | |  |  |  |
| 0 | | **1.00** |  |  |  |
| 1 | | 1.26 | (0.87 | - | 1.82) |
| 2 | | 1.28 | (0.80 | - | 2.04) |
| 3 | | 1.02 | (0.55 | - | 1.87) |
| **Antidiabetic agents** | |  |  |  |  |
| No | | **1.00** |  |  |  |
| Yes | | 0.99 | (0.63 | - | 1.56) |
| **Antihypertensive agents** | |  |  |  |  |
| No | | **1.00** |  |  |  |
| Yes | | 1.12 | (0.80 | - | 1.55) |
| **Lipid-lowering agents** | |  |  |  |  |
| No | | **1.00** |  |  |  |
| Yes | | 0.34 | (0.15 | - | 0.77) |
| **Antidepressive agents** | |  |  |  |  |
| No | | **1.00** |  |  |  |
| Yes | | 2.28 | (1.63 | - | 3.20) |
| **Malignant neoplasm** | |  |  |  |  |
| No | | **1.00** |  |  |  |
| Yes | | 0.83 | (0.33 | - | 2.10) |
| **Ischemic heart disease** | |  |  |  |  |
| No | | **1.00** |  |  |  |
| Yes | | 0.60 | (0.31 | - | 1.15) |
| **Stroke** | |  |  |  |  |
| No | | **1.00** |  |  |  |
| Yes | | 0.86 | (0.46 | - | 1.59) |

CI, confidence interval.
